# Delayed Diagnosis of Alpha-1 Antitrypsin Deficiency in an Elderly Patient

**DOI:** 10.3390/diagnostics16091329

**Published:** 2026-04-28

**Authors:** Beatrice Ragnoli, Patrizia Pochetti, Xheni Veselagu, Mario Malerba

**Affiliations:** 1 Department of Translational Medicine, University of Piemonte Orientale, 28100 Novara, Italy; beatrice.ragnoli@uniupo.it; 2 Respiratory Unit, S. Andrea Hospital, 13100 Vercelli, Italy; patrizia.pochetti@aslvc.piemonte.it (P.P.); xheni.veselagu@aslvc.piemonte.it (X.V.)

**Keywords:** alpha-1 antitrypsin deficiency, chronic obstructive pulmonary disease, pulmonary emphysema, delayed diagnosis, PI*ZZ genotype

## Abstract

**Background and Clinical Significance:** Alpha-1 antitrypsin deficiency (AATD) is an autosomal codominant disorder caused by pathogenic variants in the *SERPINA1* gene, resulting in reduced circulating alpha-1 antitrypsin (AAT) or production of dysfunctional protein. AAT is the principal inhibitor of neutrophil elastase, and its deficiency leads to unchecked proteolytic activity, progressive destruction of lung parenchyma, and increased susceptibility to infections. Severe deficiency, particularly in individuals homozygous for the Z allele (PI*ZZ), predisposes to early-onset panacinar emphysema, chronic airflow obstruction, and liver disease. Despite its clinical relevance, AATD remains markedly underdiagnosed and is frequently misclassified as smoking-related chronic obstructive pulmonary disease (COPD), delaying access to disease-modifying therapy, genetic counselling, and preventive strategies. Early recognition is therefore essential to improve outcomes. **Case Presentation:** We report the case of a 68-year-old ex-smoker with a long-standing diagnosis of “COPD” who presented with acute-on-chronic type 2 respiratory failure and community-acquired pneumonia. Spirometry revealed severe airflow obstruction, and high-resolution computed tomography demonstrated extensive basilar panlobular emphysema, raising suspicion for AATD. Serum AAT concentration was critically low at 26.8 mg·dL^−1^, and isoelectric focusing confirmed a PI*ZZ phenotype. Next-generation sequencing identified homozygosity for the *SERPINA1* c.1096G>A (Z) variant, with no additional pathogenic alleles. Cascade family screening revealed multiple heterozygous PI*MZ relatives. Before augmentation therapy could be initiated, the patient developed severe Legionella pneumophila pneumonia with secondary bacterial superinfection, progressing to refractory septic shock and death. **Conclusions:** This case illustrates how AATD can masquerade as smoking-related COPD for years, leading to missed opportunities for timely intervention. It underscores the importance of testing all adults with COPD or refractory asthma at least once, regardless of age or smoking history. Early diagnosis enables initiation of augmentation therapy, targeted vaccination, lifestyle modification, and genetic counselling, ultimately improving prognosis and reducing preventable morbidity and mortality.

## 1. Introduction

Alpha-1 antitrypsin deficiency (AATD) is an autosomal codominant disorder caused by pathogenic variants in the *SERPINA1* gene. AAT is the principal endogenous inhibitor of neutrophil elastase; its deficiency impairs the protease–antiprotease balance, resulting in progressive destruction of alveolar structures and chronic airway inflammation [1,2]. Severe deficiency is typically associated with PI*ZZ homozygosity, whereas intermediate deficiency occurs in heterozygous states such as PI*MZ and PI*SZ [3].

Although AATD was first described in 1963 by Laurell and Eriksson [4], it remains markedly under-recognized worldwide. Epidemiological studies indicate that 1–2% of all COPD cases are attributable to AATD [2], and the estimated prevalence of PI*ZZ homozygosity is approximately 1 in 2000–5000 individuals of Northern European ancestry [1]. Yet only a minority of affected individuals receive a timely diagnosis. This underdiagnosis reflects the clinical overlap with smoking-related COPD, limited clinician awareness, variability in symptom onset, and inconsistent implementation of screening recommendations, with many patients diagnosed only after irreversible lung damage has occurred.

The clinical manifestations of AATD are heterogeneous and influenced by genotype, environmental exposures, and lifestyle factors. Respiratory symptoms typically develop in the third to fifth decades and include exertional dyspnea, chronic cough, and recurrent bronchitis [3]. Radiologically, AATD is characterized by basal-predominant panlobular emphysema, which helps distinguish it from smoking-related centrilobular emphysema [5,6]. Cigarette smoking dramatically accelerates disease progression and reduces survival [7].

Diagnosis relies on a combination of biochemical and genetic testing. Serum AAT concentrations below 100 mg·dL^−1^ suggest deficiency, but levels may fluctuate during acute inflammation; therefore, confirmatory genotyping or sequencing is required to identify the specific allele combination [8]. Advances in molecular diagnostics, including next-generation sequencing, have improved the ability to detect rare or null variants that may be missed by traditional phenotyping, offering a more comprehensive genetic characterization [9].

International guidelines recommend measuring AAT levels in all adults with COPD or poorly controlled asthma at least once during their evaluation [10]. This recommendation reflects the fact that AATD is one of the few genetic respiratory disorders for which a disease-modifying therapy exists. Augmentation therapy with weekly intravenous infusions of purified human AAT (60 mg·kg^−1^) is the only approved treatment that directly addresses the underlying biochemical defect. It slows the loss of lung density on CT and has been associated with improved survival in multinational registry analyses [10,11]. In addition to augmentation therapy, comprehensive management includes smoking cessation, vaccinations, inhaled bronchodilators, pulmonary rehabilitation, long-term oxygen therapy, and, in selected cases, lung transplantation. Emerging therapeutic approaches—such as small-molecule correctors of misfolded Z-AAT, RNA interference strategies, and gene therapy—are under active investigation and may further expand treatment options in the coming years [12]. Despite these technological advances, diagnostic delay remains substantial, averaging 8–10 years [13], and continues to deprive patients of timely intervention.

The phenotypic spectrum of AATD is broader than traditionally appreciated. While the classical presentation involves a young, non-smoking individual with basal emphysema, the published case literature highlights a remarkable heterogeneity [14]. Reports describe organizing pneumonia mimicking malignancy [15], cystic lung disease in PI*IZ heterozygotes [16], coexistence of AATD and lung cancer [17], and severe disease in young never-smokers with novel homozygous PI*Null mutations [18]. These observations challenge the assumption that AATD presents in a predictable manner and underscore the need for heightened clinical suspicion across a broad range of respiratory presentations [19]. Importantly, even late diagnosis is not futile, as initiation of augmentation therapy and targeted preventive measures may still confer, even though for many patients, the window for intervention closes before the diagnosis is ever considered benefit [20].

The consequences of delayed diagnosis extend beyond respiratory morbidity. AATD is also associated with liver disease due to intracellular accumulation of misfolded Z-AAT in hepatocytes, leading to neonatal cholestasis, chronic hepatitis, cirrhosis, and hepatocellular carcinoma [20]. Although liver involvement is more common in childhood, adult-onset liver disease is increasingly recognized. Moreover, AATD has been linked to panniculitis [7], vasculitis [21], and other systemic manifestations, further broadening its clinical spectrum.

Given this complexity, early recognition of AATD is essential not only for initiating appropriate therapy but also for enabling cascade family screening [19]. Identifying heterozygous carriers allows for targeted counselling regarding smoking avoidance, occupational exposures, and lifestyle modifications that may mitigate long-term risk [19]. Genetic counselling also plays a crucial role in reproductive planning and in raising awareness among family members who may be asymptomatic but genetically at risk [19].

Here, we report the case of a 68-year-old man with homozygous PI*ZZ AATD who had been misclassified for more than a decade as having smoking-related COPD and ultimately died from fatal polymicrobial pneumonia before augmentation therapy could be initiated. This case highlights the clinical consequences of delayed diagnosis and reinforces the need for early recognition and proactive management strategies.

## 2. Case Presentation

A 68-year-old Caucasian man presented to the Emergency Department (ED) of the S. Andrea Hospital (Vercelli, Italy) with a two-day history of progressive dyspnea and productive cough. He was an ex-smoker with a 40 pack–year history and had been on long-term oxygen therapy (LTOT) since 2011 for severe emphysema previously labelled as COPD. Comorbidities included pulmonary hypertension and congestive heart failure. He had suffered multiple prior hospitalizations for presumed COPD exacerbations treated with systemic corticosteroids and antibiotics. There were no relevant occupational exposures. He reported no known family history of lung disease, although formal cascade testing had never been performed.

On admission, he was tachypneic (respiratory rate 28 breaths/min) with oxygen saturation of 92% on high-flow nasal cannula (FiO_2_ 60%). Arterial blood gas analysis revealed acute-on-chronic type II respiratory failure (pH 7.21; PaO_2_ 77.6 mmHg; PaCO_2_ 76.4 mmHg; bicarbonate 30 mmol·L^−1^). Serum C-reactive protein was 12 mg·dL^−1^ with mild leukocytosis.

**Figure 1:**
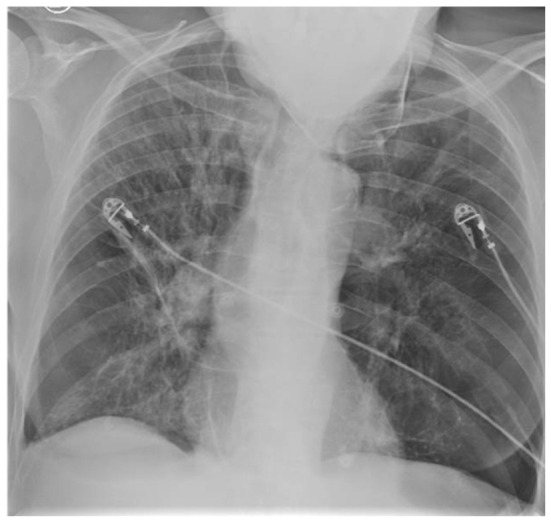
Chest radiograph obtained at presentation demonstrating hyperinflated lung fields with flattening of the diaphragms and attenuated peripheral vascular markings consistent with diffuse panlobular emphysema. A right basal consolidative infiltrate consistent with pneumonia is also present.

Chest radiography demonstrated marked bilateral hyperinflation with flattened diaphragms, attenuated peripheral vascular markings consistent with extensive emphysema, and a right basal consolidative infiltrate (Figure 1). He was therefore admitted to the pulmonary unit and commenced on non-invasive ventilation, inhaled bronchodilators, intravenous methylprednisolone, and empiric broad-spectrum antibiotics.

Given the persistence of symptoms, pulmonary function testing was performed after stabilization. Spirometry revealed severe obstructive ventilatory impairment: FEV_1_ 0.73 L (24% predicted), FVC 2.17 L (55% predicted), and FEV_1_/FVC ratio of 0.34 (44% predicted). Flows at mid-lung volumes were markedly reduced (MEF50 0.34 L·s^−1^, 9% predicted; MEF25 0.29 L·s^−1^, 43% predicted), and peak expiratory flow was 2.38 L·s^−1^ (31% predicted), consistent with severe airflow obstruction with a concave expiratory flow-volume pattern.

**Figure 2:**
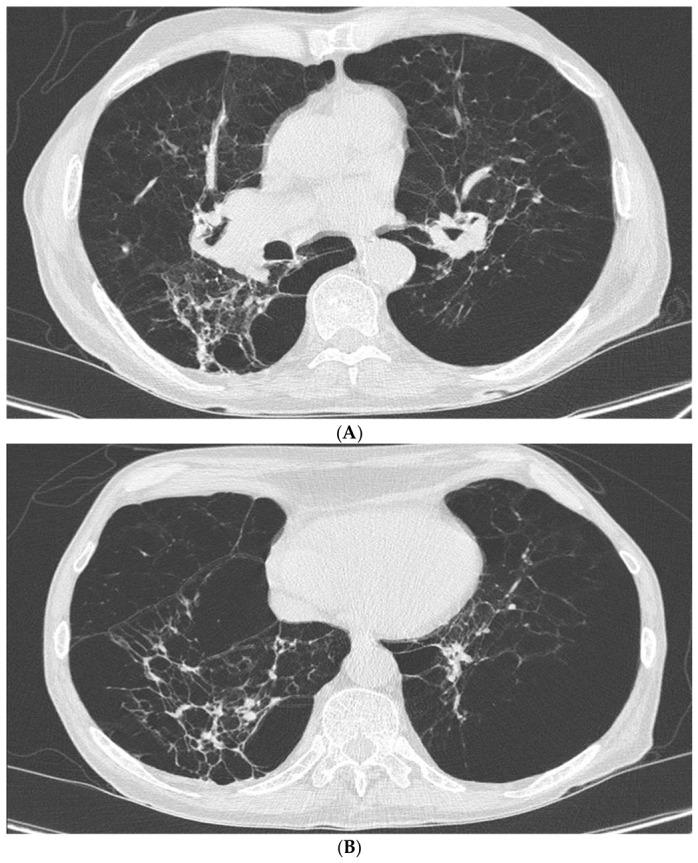
High-resolution computed tomography of the chest demonstrating extensive bilateral panlobular emphysema. (**A**) Axial section at the level of the carina showing diffuse low-attenuation areas with destruction of secondary pulmonary lobules and a right lower lobe consolidation. (**B**) Caudal axial section illustrating marked emphysematous change with thin residual alveolar septa and bronchial wall thickening.

High-resolution computed tomography (HRCT) of the chest confirmed extensive bilateral panlobular emphysema with a predominantly basal and central distribution (Figure 2A), along with a right lower lobe consolidation (Figure 2B). Sputum and blood cultures were negative. The patient improved clinically and was discharged on LTOT, oral corticosteroids, oral antibiotics, and inhaled bronchodilators, with a working diagnosis of acute-on-chronic type II respiratory failure secondary to community-acquired pneumonia.

The severity of emphysema, disproportionate to smoking history, the relatively early onset of disease, and the basal-predominant panlobular pattern on HRCT prompted investigation for AATD. Serum AAT concentration measured by nephelometry was critically low at 26.8 mg·dL^−1^ (reference range 88–174 mg·dL^−1^). Notably, this measurement was obtained when the patient had an elevated CRP (12 mg·dL^−1^); since AAT is an acute-phase reactant, the true baseline AAT level was likely even lower. Isoelectric focusing demonstrated a PI*ZZ phenotype. Confirmatory genotyping by next-generation sequencing identified homozygosity for the pathogenic SERPINA1 c.1096G>A variant (PI*Z allele), with all other tested variants returning negative (Table 1).

**Figure 3:**
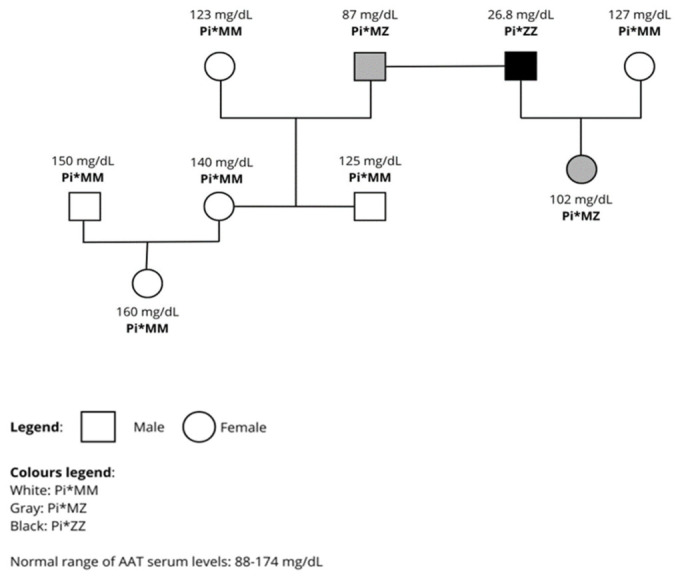
Family pedigree of the index case showing serum AAT levels and *SERPINA1* genotype for each member of the family. The proband (PI*ZZ) exhibits critically reduced AAT at 26.8 mg·dL^−1^; heterozygous PI*MZ relatives show intermediate levels; PI*MM relatives have normal serum AAT levels. Normal reference ranges:88174 mg·dL^−1^ by nephelometry.

Following the diagnosis, a three-generation family pedigree was constructed, and cascade screening was offered to first-degree relatives (Figure 3). The patient’s brother had an intermediate AAT level of 87 mg/dL and a PI*MZ genotype, indicating inheritance of one normal M allele and one deficient Z allele with intermediate AAT levels. The patient’s wife had a normal AAT level of 127 mg/dL with a PI*MM genotype. Their daughter had an AAT level of 102 mg/dL with a heterozygous PI*MZ genotype, consistent with inheritance of one deficient Z allele with intermediate AAT levels from the index case and one normal M allele from her mother. Other first-degree relatives were PI*MM with AAT levels within the normal range.

One month after discharge, the patient re-presented with severe dyspnea, fever, and purulent sputum, rapidly deteriorating despite non-invasive ventilation. He was intubated and transferred to the intensive care unit. Laboratory investigations demonstrated marked leukocytosis, C-reactive protein 33 mg·dL^−1^, and procalcitonin 7 ng·mL^−1^. Urinary antigen testing was positive for *Legionella pneumophila*, and bronchoalveolar lavage isolated *Haemophilus influenzae*, indicating a polymicrobial infection. Despite optimization of antimicrobial therapy and mechanical ventilatory support, the patient developed refractory septic shock and died on day five of admission.

## 3. Discussion

AATD remains a substantially underdiagnosed genetic disorder despite decades of scientific progress and the availability of disease-modifying therapy. The present case exemplifies the clinical consequences of delayed recognition, illustrating how AATD may be misclassified for years as smoking-related COPD when characteristic radiological and physiological features are not adequately contextualized. Our patient experienced progressive respiratory impairment beginning in his early fifties, yet the diagnosis of AATD was not established until age 68, by which time extensive panlobular emphysema, pulmonary hypertension, and chronic respiratory failure were already present. This pattern mirrors the broader epidemiological reality: more than 90% of individuals with severe AATD remain undiagnosed, and the average diagnostic delay ranges from 8 to 10 years [2]. Such delays deprive patients of timely access to disease-modifying therapy, structured preventive strategies, and family screening.

The clinical overlap between AATD and common smoking-related COPD contributes significantly to this diagnostic gap. Both conditions present with dyspnea, chronic cough, recurrent exacerbations, and airflow obstruction; however, AATD is distinguished by a characteristic basal-predominant panlobular emphysema pattern, disproportionate severity relative to smoking history, and earlier age of onset. In our patient, these features were present but remained unrecognized for over a decade. Current guidelines therefore emphasize that all adults with COPD or refractory asthma should undergo at least one lifetime measurement of serum AAT levels, regardless of age or smoking status [10]. This recommendation is particularly relevant for individuals with emphysema that is unusually severe at an early age, with basilar predominance on imaging, unexplained bronchiectasis, or a family history of liver or lung disease [2,10].

Mechanistic studies have delineated AATD as a disorder of early and genotype-specific airway vulnerability. Initial physiological evidence demonstrated heightened airway hyperresponsiveness in individuals with severe or intermediate deficiency [22]. Bronchial epithelial cells expressing the Z variant accumulate misfolded AAT polymers, activating stress pathways and amplifying inflammation [23]. Clinical studies in PI*MZ subjects identified a reproducible pattern of neutrophilic airway inflammation [24], further substantiated by distinct exhaled nitric oxide (FeNO) profiles across AATD genotypes [25]. Collectively, these insights show that AATD is characterized by early epithelial dysfunction, altered airway reactivity, and neutrophil-driven inflammation, detectable before overt airflow limitation [26], supporting the rationale for early therapeutic intervention.

In the context of these mechanistic insights, the clinical trajectory of our patient becomes even more striking. The extensive basal panlobular emphysema observed on HRCT, the severity of airflow obstruction, and the early onset of symptoms were all consistent with the pathophysiological processes described above. Early identification is critical because augmentation therapy is most effective before irreversible lung destruction has occurred. Had AATD been recognized earlier, augmentation therapy could have been initiated at a stage when lung tissue destruction was still modifiable. Evidence from multinational registries demonstrates that weekly intravenous infusion of purified human AAT (60 mg·kg^−1^) slows the decline in lung density and is associated with improved survival [11,12]. Unfortunately, by the time of diagnosis, our patient already had advanced emphysema, pulmonary hypertension, and right heart failure; his severely compromised baseline condition and subsequent severe pneumonia precluded initiation of augmentation therapy altogether. The fatal outcome starkly underscores the importance of early screening and timely preventive action.

The fatal infectious episode highlights an underrecognized aspect of AATD: increased susceptibility to severe respiratory infections. AAT deficiency impairs neutrophil elastase regulation and pathogen clearance, predisposing individuals to bacterial colonization and exaggerated inflammatory responses [23,24]. Our patient developed L. pneumophila pneumonia with polymicrobial superinfection, culminating in refractory septic shock, underscoring the importance of vaccination against influenza, pneumococcus, and COVID-19, prompt antimicrobial therapy, and, in those with frequent exacerbations and coexistent bronchiectasis, consideration of long-term macrolide therapy [27].

Beyond the individual implications of this case, the broader issue of delayed diagnosis in AATD has gained increasing attention in recent years. Several large-scale analyses have confirmed that diagnostic delay remains one of the most critical barriers to improving outcomes in this population. A European Respiratory Society expert survey involving more than 500 clinicians across 26 countries found that fewer than 40% routinely test COPD patients for AATD, despite guideline recommendations, and that lack of awareness and perceived low prevalence remain the most frequently cited barriers to testing [2]. More recent registry data from the Alpha-1 Foundation (2023) similarly highlight that the average time from first respiratory symptoms to confirmed diagnosis remains approximately 7–8 years, with many patients receiving multiple misdiagnoses—including asthma, chronic bronchitis, or “COPD of unclear etiology”—before AATD is considered [28].

The heterogeneity of AATD presentations further complicates recognition. Published case reports describe atypical manifestations, such as organizing pneumonia mimicking malignancy [15], cystic lung disease in PI*IZ genotype [16], and severe disease in young never-smokers with novel PI*Null mutations [18], collectively showcasing the breadth of clinical phenotypes associated with *SERPINA1* variants. These cases challenge the traditional view of AATD as a disease confined to young, non-smoking individuals with basal emphysema and highlight a broad phenotypic spectrum that requires clinicians to maintain a high index of suspicion across diverse clinical scenarios. In contrast to these atypical presentations, our patient’s phenotype was classic but overlooked, demonstrating that even typical cases may escape recognition when diagnostic vigilance is low.

Several studies have attempted to identify predictors of delayed diagnosis. Older age at symptom onset, smoking history, and absence of family history were independently associated with diagnostic delay, suggesting that clinicians may be less likely to suspect AATD in older or smoking individuals, even when radiological features are suggestive [29,30]. This aligns closely with our patient’s trajectory: his smoking history and age at presentation likely contributed to anchoring bias, reinforcing the assumption of smoking-related COPD and delaying appropriate testing.

Importantly, delayed diagnosis has measurable clinical consequences. Evidence from the Austrian Alpha-1 Lung Registry demonstrates that diagnostic delay is associated with significantly worse survival and more advanced disease at the time of diagnosis, underscoring the clinical impact of late recognition [31]. These findings mirror the outcome in our case, where the patient’s advanced emphysema and subsequent fatal pneumonia precluded initiation of augmentation therapy.

Recent evidence also suggests that delayed diagnosis contributes to increased healthcare utilization. A U.S. claims-based analysis demonstrated that individuals with undiagnosed AATD had significantly higher rates of emergency department visits, hospitalizations, and antibiotic prescriptions in the five years preceding diagnosis compared with matched COPD controls [32]. This underscores the economic and healthcare burden associated with missed or late recognition of AATD, reinforcing the need for systematic screening strategies.

To improve early detection, reflex testing—automatically measuring AAT levels when specific laboratory criteria are met—has shown considerable promise, increasing detection rates significantly [33]. Targeted screening of high-risk groups, including individuals with early-onset emphysema, unexplained bronchiectasis, liver disease, or panniculitis, is another effective strategy [19]. Screening first-degree relatives of confirmed cases remains one of the most efficient approaches for case finding [19]. In our case, cascade testing identified multiple PI*MZ heterozygotes, underscoring the value of family-based strategies to enable targeted counselling regarding smoking avoidance and lifestyle modifications [1,2].

The genetic analysis in this case confirmed homozygosity for the PIZ allele (PI*ZZ genotype) and excluded a comprehensive panel of rarer pathogenic *SERPINA1* variants, demonstrating the value of next-generation sequencing in establishing a definitive molecular diagnosis. The family pedigree (Figure 3) revealed the expected genotype-phenotype correlation: heterozygous PI*MZ relatives showed intermediate serum AAT levels well above the deficiency threshold, PIMM relatives had normal concentrations, while the index case (PI*ZZ) had critically low levels at 26.8 mg·dL^−1^. In our case, cascade testing identified multiple PI*MZ heterozygotes, underscoring the value of family-based strategies.

These findings reinforce the importance of cascade family screening and genetic counselling, as asymptomatic carriers (i.e., PI*MZ heterozygotes) should be advised to avoid smoking and occupational exposures, which may accelerate lung-function decline even in the heterozygous state [1,2].

Educational initiatives also play a crucial role. Recent surveys indicate that many clinicians still feel insufficiently trained to recognize atypical presentations of AATD or to interpret genetic results. In this regard, a structured survey study by Requena-Fernández et al. provides compelling quantitative evidence of this knowledge gap [34]. Administering validated questionnaires on AATD to 618 participants across four groups, comprising general pediatricians, pediatric pulmonologists, pediatric gastroenterologists, and final-year medical students, the authors found that no group reached the minimum threshold of 50% correct answers required to ensure adequate knowledge of the disease, regardless of years of clinical experience. Strikingly, even pediatric pulmonologists, who achieved the highest average score (3.12 out of 7 points), fell below this threshold. Medical students performed worst, with only 2.1% correctly identifying the clinical manifestations of AATD and 14.7% selecting the correct management options. Moreover, 70% of pediatric pulmonologists and 77% of gastroenterologists were unaware of the existence of dedicated AATD reference units, and 67.8% of all respondents did not know of the national AATD registry. These findings underscore that the knowledge deficit begins at the level of undergraduate medical education and persists across specialties, reinforcing the need to integrate AATD content into medical school curricula as a foundational step toward reducing diagnostic delay.

Targeted educational programs, online modules, and integration of AATD content into pulmonology and internal medicine curricula have been proposed as strategies to bridge this gap. In parallel, international initiatives such as the Alpha-1 Foundation, ERN-LUNG, and, more recently, the European Alpha-1 Research Collaboration (EARCO) have expanded the European AATD infrastructure by promoting education, standardized diagnostic pathways, and harmonized registry-based clinical data collection across centers. [35,36].

The rapid development of novel therapeutic platforms highlights the growing need for timely identification of AATD. Approaches such as RNA-based modulation, gene-directed interventions, and small-molecule strategies aimed at correcting Z-AAT misfolding are advancing through clinical pipelines, and their potential effectiveness will likely depend on intervention before substantial, irreversible lung injury has occurred [37,38].

This case therefore illustrates a broader principle: delayed diagnosis is not simply a missed opportunity but a modifiable determinant of long-term outcomes. Reducing diagnostic latency will require systematic screening strategies, greater clinician awareness, and the integration of automated prompts within routine care pathways.

Long-term management of AATD similarly benefits from a coordinated, multidisciplinary framework that includes behavioral measures, structured respiratory rehabilitation, optimized inhaled maintenance therapy, and, in advanced disease, evaluation for surgical interventions such as lung-volume reduction or transplantation [30]. As disease-modifying therapies continue to evolve—from agents that promote correct AAT folding to RNA-silencing and gene-replacement technologies—the therapeutic landscape is shifting toward upstream correction of the molecular defect [37]. Ensuring early recognition of affected individuals will be essential to fully realize the benefits of these emerging options.

## 4. Conclusions

AATD is a treatable but chronically under-diagnosed genetic disorder. This case of a 68-year-old man with severe panlobular emphysema demonstrates how mislabeling as smoking-related COPD can delay diagnosis for over a decade, with ultimately fatal consequences and reinforcing the need for systematic screening in all adults with COPD or refractory asthma.

Clinicians should therefore measure serum AAT levels in all patients with COPD or refractory asthma, particularly with basilar emphysema that arises at a relatively young age, or predominates at the lung bases on HRCT. Early recognition enables timely initiation of augmentation therapy, cascade family screening, and preventive measures including avoidance of tobacco and noxious exposures. Sustained awareness and consistent adherence to international screening recommendations remain essential to improving outcomes in patients with AATD.

## Figures and Tables

**Table 1:** *SERPINA1* molecular genotyping results in the index patient.

Serum AAT Levels 26.8 mg/dL Isoelectric Focusing (IEF) Z/Z		
A1AT Genotyping Test		
c.187C>T	c.194C>T	c.22G-228delTTC
Negative	Negative	Negative
PI*I	PI*Mprocida	PI*Mamlton, PI*Mpalermo, PI*Mnichinan
c.230C>T	c.551_552delC	c.647G>T
Negative	Negative	Negative
PI*Siyama	PI*Q0granite falls	PI*Q0west
c.721A>T	c.739C>T	c.839>T
Negative	Negative	Negative
PI*Q0bellingham	PI*F	PI*Plowell, PI*Pduarte, PI*Q0cardiff, PI*Ybarcelona
c.863A>T	c.1096G>A Homozygous PI*Z	c.1130_1331insT
Negative		Negative
PI*S		PI*Q0mattawa, PI*Q0ourem
c.1156_1157insC	c.1178>T	
Negative	Negative	
PI*Q0clayton, PI*Q0Saarbruecken	PI*Mheerlen	
Final Genotype PI*ZZ		

Next-generation sequencing was performed to characterize the patient’s *SERPINA1* genotype. All tested variants returned negative except for the c.1096G>A substitution (PI*Z allele), which was identified in the homozygous state, confirming a final genotype of PI*ZZ. Abbreviations: AAT, alpha-1 antitrypsin; * represents a standardized element of the protease inhibitor (PI) classification system and is used to denote the specific allelic combination at the SERPINA1 locus. Thus, the designation PI*MZ correctly identifies an individual heterozygous for the M and Z alleles*.

## Data Availability

The raw data supporting the conclusions of this article will be made available by the authors upon request.
